# Vasomotor and physical menopausal symptoms are associated with sleep quality

**DOI:** 10.1371/journal.pone.0192934

**Published:** 2018-02-20

**Authors:** Min-Ju Kim, Gyeyoon Yim, Hyun-Young Park

**Affiliations:** Division of Cardiovascular Diseases, Center for Biomedical Science, National Research Institute of Health, Chungbuk, Korea; Federal University of São Paulo, BRAZIL

## Abstract

**Background:**

Sleep disturbance is one of the common complaints in menopause. This study investigated the relationship between menopausal symptoms and sleep quality in middle-aged women.

**Method:**

This cross-sectional observational study involved 634 women aged 44–56 years attending a healthcare center at Kangbuk Samsung Hospitals. Sleep quality was measured using the Pittsburgh Sleep Quality Index (PSQI).Multiple linear regression analysis was performed to assess the associations between Menopause-specific Quality of Life (MENQOL) scores and PSQI scores and Menopause-specific Quality of Life (MENQOL)scores.

**Results:**

The mean PSQI score was 3.6±2.3, and the rates of poor sleep quality(PSQI score > 5) in premenopausal, perimenopausal, and postmenopausal women were 14.4%, 18.2%, and 30.2%, respectively. Total PSQI score, specifically the sleep latency, habitual sleep efficiency and sleep disturbances scores, were significantly increased in postmenopausal women. Multiple linear regression analysis adjusted for age, BMI, hypertension, diabetes, smoking, marital status, family income, education, employment status, parity, physical activity, depression symptoms, perceived stress and menopausal status showed that higher PSQI score was positively correlated with higher vasomotor(ß = 0.240, *P* = 0.020)and physical(ß = 0.572, *P*<0.001) scores.

**Conclusions:**

Vasomotor and physical menopause symptoms was related to poor sleep quality. Effective management strategies aimed at reducing menopausal symptoms may improve sleep quality among women around the time of menopause.

## Introduction

Sleep disorders are the most common complaints during menopause transition and postmenopause; these disorders include trouble falling asleep, waking up several times during the night, and waking up earlier than desired in the morning [1,2]. Poor sleep quality and inadequate sleep duration are associated with negative health outcomes, such as obesity, cardiovascular disease, cancer-related mortality, diabetes, depression, and poor quality of life[3–9].Therefore, it is necessary to understand sleep disorders and to identify related risk factors in menopausal women.

Several studies show that perimenopausal and postmenopausal women are more likely to report sleep disturbance than premenopausal women[10–12]. For example, a study in a large sample of Colombian women found that sleep problems in middle-aged women were associated with the severity of menopausal symptoms, tobacco use, and the presence of hypertension[9].Significant risk factors for sleep disturbance among middle-aged Chinese women include menopause status and menopausal symptoms, in addition to older age and a history of chronic disease[10].Age, menopause and related symptoms, perceived stress, mood problems, and hypertension have been identified as potential risk factors for sleep disturbance[13–16].

Despite these findings, it may be difficult to determine which factors influence sleep disturbance in menopausal women. Although hormonal changes during menopause influence sleep-related complaints, other factors may also play a significant role[1].In particular, sleep disturbance is known to interact with menopausal symptoms, especially vasomotor symptoms[17,18].A study investigating the relationship between sleep quality and menopausal symptoms found that poorer sleep quality was positively associated with higher rates of somatic and psychological symptoms, and menopausal symptoms were more common in women with frequent sleep complaints than those with infrequent sleep complaints[9,18].

Although menopausal symptoms have been associated with sleep disturbance, previous studies have focused on vasomotor symptoms. In addition, few studies have investigated the associations between the psychosocial, physical and sexual menopausal symptoms and sleep quality. Therefore, this study investigated the relationship between menopausal symptoms and self-reported sleep quality in middle-aged women.

## Materials and methods

### Study participants

We have used data from the Kangbuk Samsung Health Study. It is a cohort study of South Korean men and women aged 18 years or older who underwent a comprehensive annual or biennial health examination at the clinics of the Kangbuk Samsung Hospital Total Healthcare Center in Seoul and Suwon, South Korea. This cross-sectional observational study was designed to investigate the attitudes of Korean women towards menopause. In total, 2,204 middle-aged women aged 44 to 56 years who visited a healthcare center between November 2012 and March 2013 was selected and the participation rate was about 71%. Subjects who were diagnosed with or were being treated for serious illnesses such as cancer were excluded at the screening stage. The details of the present cohort have been described elsewhere [19].The association between sleep quality and menopausal symptoms was investigated in 950 participants who completed a sleep questionnaire. Of these, 171 subjects with a history of hormone replacement therapy for the management of menopausal symptoms, and 145 with missing data on the Menopause-specific Quality of Life (MENQOL)questionnaire were excluded from analysis. Therefore, 634 women were eligible for this study. The study was approved by the Institutional Review Board of Kangbuk Samsung Hospital (IRB No. KBC12156).Written informed consent was obtained from all subjects prior to participation in this study.

### Measurements

Body mass index (BMI) was calculated as body weight in kilograms divided by the square of height in meters. Waist circumference (WC) was measured at the midpoint between the lower ribs and the top of the iliac crest in the standing position. Blood pressure (BP) was measured three times using a Welch Allyn sphygmomanometer after a 5min rest period, and the final systolic and diastolic BP were each calculated from two measurements.

Smoking status was categorized as non-current or current. Marital status was categorized as living without or with a partner. Family income was classified as < 4 million won and> 4 million won per month. Educational level was categorized as high school or lower, or college/university. Working status was categorized as employed or unemployed. Parity was characterized as 1–2 children and 3 or more children.

Depressive symptoms were assessed using the Korean version of the Center for Epidemiologic Studies Depression Scale (CES-D);CES-D scores≥ 16 were regarded as indicating depressive symptoms[20,21].

Perceived stress was measured using the stress questionnaire for Korean National Health and Nutrition Examination Survey (KNHANES) [22]. Higher scores indicate a higher level of stress.

Menopausal status was defined using the Stages of Reproductive Aging Workshop(STRAW) criteria. Women having regular menstrual periods were defined as premenopausal; those with persistent differences of more than 7 days in the length of consecutive cycles or amenorrhea lasting more than 60 days were regarded as perimenopausal; and women with at least 12 months of amenorrhea classified as postmenopausal[23].

### Menopausal symptoms

The MENQOL questionnaire was used to assess menopause-related symptoms. The 29-item MENQOL is divided into four scales, assessing vasomotor (three items), psychosocial (seven items), physical (16 items) and sexual (three items) domains, with the score on each item ranging from 1 (not experiencing a symptom) to 8 (extremely bothered)[24].

### Sleep quality assessment

The Pittsburgh Sleep Quality Index (PSQI) is a self-rated questionnaire that measures sleep quality during the previous month[25]. It consists of 19 items, grouped into seven components: subjective sleep quality, sleep latency, sleep duration, habitual sleep efficiency, sleep disturbance, use of sleeping medication, and daytime dysfunction. Each item has a score ranging from 0 to 3; the seven component scores are summed to yield a global PSQI score, which ranges from 0 to 21. Sleep quality was categorized as good (PSQI≤5) or poor (PSQI>5), with higher PSQI scores indicating poorer sleep quality.

### Statistical analysis

Data were expressed as mean ± standard deviation (SD) or number (%).Normal distribution was tested using the Shapiro-Wilk test, and the data is normally distributed. Differences between groups were compared using independent t-tests for parametric data, and non-parametric data compared using the Mann-Whitney *U* test or Kruskal-Wallis test. Categorical variables were compared using the chi-square test. Multiple linear regression analysis was performed to assess the relationship between MENQOL scores and PSQI scores after adjustments for age, BMI, hypertension, diabetes, smoking, marital status, family income, education, employment status, parity, physical activity, depression symptoms, perceived stress and menopausal status. *P-*values <0.05 were considered statistically significant. All data were analyzed using SPSS Statistics 22 (SPSS Inc., IBM Corp., Chicago, IL, USA).

## Results

### Characteristics of the study population

The demographic characteristics of the study participants are shown in Table 1. The mean age of the participants was48.3±3.3 years, and their mean BMI was 23.1±3.1 kg/m^2^.The mean PSQI score among all subjects was 3.6±2.3, and 19.4% of the subjects reported poor sleep quality(PSQI score >5). The rates of poor sleep quality in premenopausal, perimenopausal, and postmenopausal women were 14.4%, 18.2%, and 30.2%, respectively. Diastolic BP, total cholesterol (TC), low-density lipoprotein cholesterol (LDL-C), and triglyceride (TG) levels were significantly higher in subjects with poor sleep quality. Compared with women with good sleep quality, those with poor sleep quality were slightly older, non-smokers, more likely to be living without a partner, less educated, and more likely to have depressive symptoms and higher level of stress.

**Table 1:** Demographic characteristics of the study population.

Variables	Total (n = 634)			PSQI						*P*-value
				≤ 5 (n = 511)			> 5 (n = 123)			
Age, years	48.3	±	3.3	48.1	±	3.2	49.2	±	3.6	0.003
BMI, kg/m^2^	23.1	±	3.1	23.0	±	3.0	23.5	±	3.2	0.093
WC, cm	79.0	±	7.9	78.6	±	7.8	80.3	±	8.0	0.036
SBP (mmHg)	105.1	±	13.3	104.8	±	13.3	106.3	±	13.0	0.268
DBP (mmHg)	68.3	±	10.0	67.8	±	10.1	70.3	±	9.7	0.014
FPG (mg/dL)	95.4	±	15.4	95.2	±	15.9	96.3	±	13.4	0.458
TC (mg/dL)	200.7	±	33.0	199.2	±	31.4	206.9	±	38.3	0.040
HDL-C (mg/dL)	64.2	±	14.9	64.5	±	15.0	63.0	±	14.8	0.341
LDL-C (mg/dL)	122.0	±	30.3	120.5	±	28.7	128.0	±	35.8	0.032
TG (mg/dL)	93.1	±	52.3	90.9	±	49.6	102.3	±	61.3	0.029
Smoking, n (%)	16	(3.3)		10	(2.5)		6	(7.0)		0.037
Marital status, n (%)										0.003
Never-married/separated/divorced/widowed	55	(8.8)		36	(7.1)		19	(15.7)		
Married/cohabitating	572	(91.2)		470	(92.9)		102	(84.3)		
Family income, n (%)										0.046
Less than 4 million won	67	(12.5)		48	(11.1)		19	(18.3)		
More than 4 million won	471	(87.5)		386	(88.9)		85	(81.7)		
Education, n (%)										<0.001
High school or lower	205	(33.3)		145	(29.1)		60	(50.8)		
College/university	411	(66.7)		353	(70.9)		58	(49.2)		
Employment status										0.654
Yes	271	(46.9)		222	(47.3)		49	(45.0)		
No	307	(53.1)		247	(52.7)		60	(55.0)		
Parity, n (%)										0.721
1–2	495	(80.9)		399	(80.6)		96	(82.1)		
≥ 3	117	(19.1)		96	(19.4)		21	(17.9)		
Physical activity										0.206
Low	361	(57.0)		283	(55.5)		78	(63.4)		
Moderate	201	(31.8)		170	(33.3)		31	(25.2)		
High	71	(11.2)		57	(11.2)		14	(11.4)		
Depressive symptoms (CES-D)										<0.001
< 16	529	(89.1)		444	(92.9)		85	(73.3)		
≥ 16	65	(10.9)		34	(7.1)		31	(26.7)		
Perceived stress scores	17.4	±	16.7	14.5	±	14.6	28.9	±	19.3	<0.001
Menopausal status										<0.001
Premenopause	271	(42.7)		232	(45.4)		39	(31.7)		
Perimenopause	214	(33.8)		175	(34.2)		39	(31.7)		
Postmenopause	149	(23.5)		104	(20.4)		45	(36.6)		
Total MENQOL score	2.8	±	1.26	2.6	±	1.15	3.5	±	1.40	<0.001
Total PSQI score	3.6	±	2.29	2.7	±	1.33	7.3	±	1.61	<0.001

Data are expressed as mean ± standard deviation or number (%).

Between group comparisons of continuous variables were analyzed by independent sample *t*-tests, and comparisons of categorical variables by chi-square tests.

BMI, body mass index; WC, waist circumference; SBP, systolic blood pressure; DBP, diastolic blood pressure; FPG, fasting plasma glucose; TC, total cholesterol; HDL-C, high-density lipoprotein cholesterol; LDL-C, low-density lipoprotein cholesterol; TG, triglyceride CES-D, the Center for Epidemiology Studies Depression scale; MENQOL, Menopause-specific Quality of Life; PSQI, Pittsburgh Sleep Quality Index

### Total PSQI and subscale scores

Table 2 presents the total PSQI and subscale scores according to menopausal status. Total PSQI scores were significantly higher in postmenopausal than in premenopausal women(4.14±2.50 vs. 3.26±2.03,*P* = 0.002). Several of the PSQI subscale scores, specifically sleep latency, habitual sleep efficiency and sleep disturbances scores, also differed significantly according to menopausal status. Sleep duration was the most highly rated of all items, but did not differ significantly among the three menopausal stages.

**Table 2:** Total Pittsburgh Sleep Quality Index (PSQI) and subscale scores according to menopausal status.

PSQI items	Menopausal status									*P*-value
	Premenopause (n = 271)			Perimenopause (n = 214)			Postmenopause (n = 149)			
Subjective sleep quality	0.40	±	0.59	0.41	±	0.60	0.51	±	0.64	0.160
Sleep latency	0.56	±	0.78	0.65	±	0.85	0.86	±	0.89	0.001
Sleep duration	1.08	±	0.65	1.21	±	0.75	1.15	±	0.70	0.220
Habitual sleep efficiency	0.11	±	0.42	0.14	±	0.49	0.24	±	0.61	0.040
Sleep disturbances	0.57	±	0.50	0.68	±	0.55	0.70	±	0.47	0.018
Use of sleep medication	0.00	±	0.06	0.02	±	0.17	0.05	±	0.36	0.122
Daytime dysfunction	0.54	±	0.66	0.58	±	0.73	0.62	±	0.68	0.475
Total PSQI score	3.26	±	2.03	3.69	±	2.37	4.14	±	2.50	0.002

Data are expressed as mean ± standard deviation and compared by Kruskal-Wallis tests.

Higher total PSQI and subscale scores indicate poorer quality of sleep.

### Correlation between MENQOL and PSQI scores

Table 3 shows correlations between MENQOL and PSQI scores. MENQOL subscale scores showed significant correlations with total PSQI score (P<0.001). Vasomotor symptom correlated positively with all items on the PSQI, and the association was strongest with sleep disturbances (r = 0.243, P<0.001). All PSQI items except habitual sleep efficiency were significantly associated with psychosocial symptoms. Physical symptom correlated positively with all PSQI items except habitual sleep efficiency and use of sleep medication. Among all PSQI items, subjective sleep quality had the highest correlation with psychosocial (r = 0.291, P<0.001) and physical symptoms (r = 0.261, P<0.001). Overall, sexual symptom had the lowest correlation coefficient of all PSQI items. The correlation between MENQOL and PSQI scores by menopausal status was showed in S1 Table.

**Table 3:** Correlations between MENQOL subscale scores and PSQI (total and subscale) scores.

PSQI items	MENQOL			
	Vasomotor	Psychosocial	Physical	Sexual
Subjective sleep quality	0.145**	0.291**	0.261**	0.141**
Sleep latency	0.231**	0.208**	0.227**	0.116*
Sleep duration	0.099*	0.095*	0.119*	0.077
Habitual sleep efficiency	0.110*	0.061	0.058	0.023
Sleep disturbances	0.243**	0.209**	0.249**	0.143**
Use of sleep medication	0.080*	0.088*	0.029	0.048
Daytime dysfunction	0.140**	0.263**	0.243**	0.078*
Total PSQI score	0.260**	0.334**	0.328**	0.160**

*P<0.05

**P<0.001.

### Association between menopausal symptoms and sleep quality

Table 4 shows the results of multiple linear regression analysis of the relationship between menopausal symptoms and sleep quality. Higher PSQI score was positively associated with higher vasomotor (ß = 0.240, *P* = 0.020) and physical (ß = 0.572, *P*<0.001) scores on the MENQOL, after adjustment for age, BMI, hypertension, diabetes, smoking, marital status, family income, education, employment status, parity, physical activity, depression symptoms, perceived stress and menopausal status. Of the four MENQOL domains, physical score showed the strongest positive association with total PSQI score. However, no association were observed between psychosocial and sexual score and total PSQI score. The overall regression model for the MENQOL subscale scores explained 28.2 percent of the variance in Total PSQI score.

**Table 4:** Multiple linear regression analysis of the relationship between MENQOL subscale scores and total PSQI score.

MENQOL		Total PSQI score			
	ß	SE	beta	*P*-value	Adjusted-R^2^
Vasomotor	0.240	0.103	0.146	0.020	0.282
Psychosocial	-0.118	0.147	-0.074	0.422	
Physical	0.572	0.153	0.307	<0.001	
Sexual	-0.131	0.075	-0.105	0.082	

Beta coefficients and *P*-values are presented.

Adjusted for age, body mass index, hypertension, diabetes, smoking, marital status, family income, education, employment status, parity, physical activity, depression symptoms (CES-D), perceived stress symptoms and menopausal status.

## Discussion

The present study examined the relationship between self-reported menopausal symptoms and sleep quality in healthy women aged 44–56 years, after controlling for potential confounders. Total PSQI score and subscores for sleep latency, habitual sleep efficiency and sleep disturbances differed significantly according to menopausal status. Poor sleep quality was related with vasomotor and physical menopausal symptoms but not with psychosocial and sexual symptoms. In particular, physical symptom showed the strongest association with sleep quality.

The prevalence of poor sleep quality differs among ethnic groups, being lower in Asian populations[11]. A cross-sectional study found that almost 40% of women aged 40–55 years reported sleep difficulty, as assessed by a single question on sleep difficulty; however ethnicity-stratified results showed that Caucasian women (40.3%) had the highest rate of sleep difficulty, and Japanese (28.2%) and Chinese (31.6%) women had the lowest rates[11]. Results from a community-based sample in Hong Kong found that about 26% of the women were poor sleepers as defined by a PSQI score > 5[26]. A large scale cross-sectional study reported that 18.1% of Chinese women aged 45–65 years suffered from insomnia[27]. Consistent with previous findings, our results showed that about 19% of the women reported poor sleep quality, and that poor sleep quality was more common in postmenopausal (30.2%) than in premenopausal women (14.4%).

Most earlier studies found that menopausal status was significantly associated with poor sleep quality[11,14,26]. In particular, a previous study in Korea found that insomnia was significantly associated with the menopausal transition[28].Our results showed that the prevalence of poor sleep quality was significantly higher in postmenopausal than in premenopausal women. In addition, total PSQI score increased according to menopause status, as did several PSQI items, including sleep latency, habitual sleep efficiency and sleep disturbances.

In addition to menopausal status, we found that poor sleep quality was highly prevalent in women with lower education and income levels. These findings were consistent with results showing that the rate of sleep disturbance among middle-aged women decreased with increasing education and income levels [10,29].Therefore, our results may indicate that higher education and income levels had a favorable influence on the sleep quality.

Previous studies also report associations between menopausal symptoms and sleep disturbances [9,10,18]. Our study found that women with higher total MENQOL and subscale scores had higher total PSQI scores, indicating that women with more severe menopausal symptoms experienced poorer sleep quality. Interestingly, our multiple linear regression models showed that vasomotor and physical symptoms, but not psychosocial and sexual symptoms, on the MENQOL were significantly related to sleep quality after adjusting for confounding factors, independent of menopause status. Of the four MENQOL domains, physical symptoms showed the highest correlation with sleep quality. In multiple linear regression analysis of Table 4, the income (ß = 1.045, *P* = 0.004), education (ß = -1.106, *P*<0.001), and depression symptoms (ß = 0.065, *P* = 0.002) were significantly associated with the overall PSQI. This might be influenced sleep disturbance. Consistent with our findings, previous studies in middle-aged women showed that sleep problems were associated with somatic and psychological menopausal symptoms[9,26]. In addition, a cross-sectional study of postmenopausal women attending a menopause clinic found that vasomotor symptoms were related with various symptoms of sleep disturbance[30]. By contrast, a study in middle-aged Chinese women reported no association between vasomotor symptoms and sleep disturbance[26].

The causes of sleep disturbance during menopause are unclear, but many factors may be involved, including vasomotor symptoms, changing hormone levels, mood disorders, coexistent medical conditions, and lifestyle factors [31].In particular, reduced hormone levels may have a significant impact on sleep disturbance. Studies have shown that hormone replacement therapy improved the sleep quality in peri- and postmenopausal women[32–35]. The beneficial effect of hormone therapy may be due to the presence of estrogen and estrogen receptors in the central nervous system, which is involved in sleep regulation[36–37].In addition, obesity may have a negative impact on sleep quality, as was shown by results from Finland on postmenopausal women, which reported impaired sleep quality in women with high BMI [18]. High BMI predisposes to partial upper-airway obstruction and increased respiratory resistance, which may contribute to poorer sleep quality[38,39]. Therefore, longitudinal studies are needed to better understand the association between these factors and sleep disturbance. Although our findings require confirmation, they provide evidence regarding the associations between menopausal symptoms and sleep quality in middle-aged women.

To our knowledge, this is one of few studies to evaluate the relationship between menopausal symptoms and sleep quality in Asian women using validated instruments. However, this study had several limitations. First, its cross-sectional design prevented the determination of the causality of the relationship, indicating the need for longitudinal studies to determine the association between menopausal symptoms and sleep quality. Second, our study population consisted of middle-aged healthy women attending a healthcare center. Thus, these results may not be applicable to the entire menopausal population in Korea. Finally, sleep quality and menopausal symptoms were measured using self-reported questionnaires, suggesting the need for objective assessments, such as polysomnography.

## Conclusions

Vasomotor and physical menopausal symptoms was related to poor sleep quality. Although further prospective studies are required to better understand these findings, effective management strategies aimed at reducing menopausal symptoms may improve sleep quality.

## Supporting information

S1 Table Correlations between MENQOL and PSQI scores by menopausal status. (DOCX)

## References

[1] Guidozzi F. Sleep and sleep disorders in menopausal women. Climacteric. 2013;16:214–219. doi: 10.3109/13697137.2012.753873 23205646 10.3109/13697137.2012.753873

[2] Owens JF, Matthews KA. Sleep disturbance in healthy middle-aged women. Maturitas.1998;30:41–50. 9819782 10.1016/s0378-5122(98)00039-5

[3] Prather AA, Puterman E, Epel ES, Dhabhar FS. Poor sleep quality potentiates stress-induced cytokine reactivity in postmenopausal women with high visceral abdominal adiposity. Brain Behav Immun.2014;35:155–162. doi: 10.1016/j.bbi.2013.09.010 24060585 10.1016/j.bbi.2013.09.010 PMC3962521

[4] Sands-Lincoln M, Loucks EB, Lu B, Carskadon MA, Sharkey K, Stefanick ML, et al Sleep duration, insomnia, and coronary heart disease among postmenopausal women in the Women's Health Initiative. J Womens Health (Larchmt).2013;22:477–486. 23651054 10.1089/jwh.2012.3918 PMC3678565

[5] Gallicchio L, Kalesan B. Sleep duration and mortality: a systematic review and meta-analysis. J Sleep Res.2009;18:148–158. doi: 10.1111/j.1365-2869.2008.00732.x 19645960 10.1111/j.1365-2869.2008.00732.x

[6] Cappuccio FP, Cooper D, D'Elia L, Strazzullo P, Miller MA. Sleep duration predicts cardiovascular outcomes: a systematic review and meta-analysis of prospective studies. Eur Heart J.2011;32:1484–1492. doi: 10.1093/eurheartj/ehr007 21300732 10.1093/eurheartj/ehr007

[7] Regestein Q, Natarajan V, Pavlova M, Kawasaki S, Gleason R, Koff E. Sleep debt and depression in female college students. Psychiatry Res. 2010;176:34–39. doi: 10.1016/j.psychres.2008.11.006 20079935 10.1016/j.psychres.2008.11.006

[8] Baker FC, Wolfson AR, Lee KA. Association of sociodemographic, lifestyle, and health factors with sleep quality and daytime sleepiness in women: findings from the 2007 National Sleep Foundation "Sleep in America Poll". J Womens Health (Larchmt).2009;18:841–849. 19514826 10.1089/jwh.2008.0986

[9] Monterrosa-Castro A, Marrugo-Flórez M, Romero-Pérez I, Fernández-Alonso AM, Chedraui P, Pérez-López FR. Assessment of sleep quality and correlates in a large cohort of Colombian women around menopause. Menopause.2013;20:464–469. doi: 10.1097/gme.0b013e31826e7649 23096246 10.1097/gme.0b013e31826e7649

[10] Sun D, Shao H, Li C, Tao M. Sleep disturbance and correlates in menopausal women in Shanghai. J Psychosom Res.2014;76:237–241. doi: 10.1016/j.jpsychores.2013.12.002 24529044 10.1016/j.jpsychores.2013.12.002

[11] Kravitz HM, Ganz PA, Bromberger J, Powell LH, Sutton-Tyrrell K, Meyer PM. Sleep difficulty in women at midlife: a community survey of sleep and the menopausal transition. Menopause.2003;10:19–28. 12544673 10.1097/00042192-200310010-00005

[12] Kuh DL, Wadsworth M, Hardy R. Women's health in midlife: the influence of the menopause, social factors and health in earlier life. Br J ObstetGynaecol.1997;104:923–933. 10.1111/j.1471-0528.1997.tb14352.x 9255084

[13] Cuadros JL, Fernández-Alonso AM, Cuadros-Celorrio AM, Fernández-Luzón N, Guadix-Peinado MJ, del Cid-Martín N, et al Perceived stress, insomnia and related factors in women around the menopause. Maturitas.2012;72:367–372. doi: 10.1016/j.maturitas.2012.05.012 22721806 10.1016/j.maturitas.2012.05.012

[14] Cheng MH, Hsu CY, Wang SJ, Lee SJ, Wang PH, Fuh JL. The relationship of self-reported sleep disturbance, mood, and menopause in a community study. Menopause.2008;15:958–962. doi: 10.1097/gme.0b013e318160dafa 18779680 10.1097/gme.0b013e318160dafa

[15] Seib C, Anderson D, Lee K. Prevalence and correlates of sleep disturbance in postmenopausal women: the Australian Healthy Aging of Women (HOW) Study. J Womens Health (Larchmt).2014;23:151–158. 24261649 10.1089/jwh.2013.4472

[16] Stranges S1, Dorn JM, Cappuccio FP, Donahue RP, Rafalson LB, Hovey KM, et al A population-based study of reduced sleep duration and hypertension: the strongest association may be in premenopausal women. J Hypertens.2010;28:896–902. doi: 10.1097/HJH.0b013e328335d076 20040890 10.1097/HJH.0b013e328335d076

[17] Moreno-Frías C, Figueroa-Vega N, Malacara JM. Relationship of sleep alterations with perimenopausal and postmenopausal symptoms. Menopause.2014;21:1017–1022. doi: 10.1097/GME.0000000000000206 24569619 10.1097/GME.0000000000000206

[18] Polo-Kantola P, Erkkola R, Irjala K, Helenius H, Pullinen S, Polo O. Climacteric symptoms and sleep quality. Obstet Gynecol.1999;94:219–224. 10432131 10.1016/s0029-7844(99)00284-7

[19] Kim MJ, Cho J, Ahn Y, Yim G, Park HY. Association between physical activity and menopausal symptoms in perimenopausal women. BMC Womens Health.2014;14:122 doi: 10.1186/1472-6874-14-122 25277534 10.1186/1472-6874-14-122 PMC4287540

[20] Radloff Lenore Sawyer. The CES-D scale a self-report depression scale for research in the general population. Applied psychological measurement.1977;1:385–401.

[21] Cho MJ, Kim KH. Use of the Center for Epidemiologic Studies Depression (CES-D) Scale in Korea. J NervMent Dis.1998;186:304–310. 10.1097/00005053-199805000-00007 9612448

[22] Shin HC. Measuring stress with questionnaires. J Korean Med Assoc. 2013;56: 485–495.

[23] Harlow SD, Gass M, Hall JE, Lobo R, Maki P, Rebar RW, et al Executive summary of the Stages of Reproductive Aging Workshop +10: addressing the unfinished agenda of staging reproductive aging. Climacteric.2012;15:105–114. doi: 10.3109/13697137.2011.650656 22338612 10.3109/13697137.2011.650656 PMC3580996

[24] Hilditch JR, Lewis J, Peter A, van Maris B, Ross A, Franssen E, et al A menopause-specific quality of life questionnaire: development and psychometric properties. Maturitas.2008;61:107–121. 19434884 10.1016/j.maturitas.2008.09.014

[25] Buysse DJ, Reynolds CF 3rd, Monk TH, Berman SR, Kupfer DJ. The Pittsburgh Sleep Quality Index: a new instrument for psychiatric practice and research. Psychiatry Res.1989;28:193–213. 2748771 10.1016/0165-1781(89)90047-4

[26] Chung KF, Tang MK. Subjective sleep disturbance and its correlates in middle-aged Hong Kong Chinese women. Maturitas.2006;53:396–404. doi: 10.1016/j.maturitas.2005.07.001 16107305 10.1016/j.maturitas.2005.07.001

[27] Li RH, Wing YK, Ho SC, Fong SY. Gender differences in insomnia—a study in the Hong Kong Chinese population. J Psychosom Res.2002;53:601–609. 12127178 10.1016/s0022-3999(02)00437-3

[28] Shin C, Lee S, Lee T, Shin K, Yi H, Kimm K, et al Prevalence of insomnia and its relationship to menopausal status in middle-aged Korean women. Psychiatry ClinNeurosci.2005;59:395–402. 10.1111/j.1440-1819.2005.01391.x 16048444

[29] Blümel JE, Cano A, Mezones-Holguín E, Barón G, Bencosme A, Benítez Z, et al A multinational study of sleep disorders during female mid-life. Maturitas.2012;72:359–366. doi: 10.1016/j.maturitas.2012.05.011 22717489 10.1016/j.maturitas.2012.05.011

[30] Vousoura E, Spyropoulou AC, Koundi KL, Tzavara C, Verdeli H, Paparrigopoulos T, et al Vasomotor and depression symptoms may be associated with different sleep disturbance patterns in postmenopausal women. Menopause.2015;22:1053–1057. doi: 10.1097/GME.0000000000000442 25783470 10.1097/GME.0000000000000442

[31] Ameratunga D, Goldin J, Hickey M. Sleep disturbance in menopause. Intern Med J.2012;42:742–747. doi: 10.1111/j.1445-5994.2012.02723.x 22288870 10.1111/j.1445-5994.2012.02723.x

[32] Schiff I, Regestein Q, Tulchinsky D, Ryan KJ. Effects of estrogens on sleep and psychological state of hypogonadal women. JAMA.1979;242:2405–2407. 226735

[33] Thomson J, Oswald I. Effect of oestrogen on the sleep, mood, and anxiety of menopausal women. Br Med J. 1977;2:1317–1319. 338104 10.1136/bmj.2.6098.1317 PMC1632495

[34] Moe KE, Larsen LH, Vitiello MV, Prinz PN. Estrogen replacement therapy moderates the sleep disruption associated with nocturnal blood sampling. Sleep.2001;24:886–894. 11766158 10.1093/sleep/24.8.886

[35] Sarti CD, Chiantera A, Graziottin A, Ognisanti F, Sidoli C, Mincigrucci M, et al Hormone therapy and sleep quality in women around menopause. Menopause.2005;12:545–551. doi: 10.1097/01.gme.0000172270.70690.5e 16145308 10.1097/01.gme.0000172270.70690.5e

[36] Krystal AD, Edinger J, Wohlgemuth W, Marsh GR. Sleep in peri-menopausal and post-menopausal women. Sleep Med Rev.1998;2:243–253. 15310495 10.1016/s1087-0792(98)90011-9

[37] Polo-Kantola P, Erkkola R, Helenius H, Irjala K, Polo O. When does estrogen replacement therapy improve sleep quality? Am J Obstet Gynecol.1998;178:1002–1009. 9609575 10.1016/s0002-9378(98)70539-3

[38] Polo. Partial upper airway obstruction during sleep. Studies with the static charge-sensitive bed (SCSB). ActaPhysiolScand Suppl. 1992;145:1–118. 1502909

[39] Guilleminault C, Quera-Salva MA, Partinen M, Jamieson A. Women and the obstructive sleep apnea syndrome. Chest. 1988;93:104–109. 3335138 10.1378/chest.93.1.104

