# Supplementary material for: Vasomotor and physical menopausal symptoms are associated with sleep quality
Source: PLoS One. 2018 Feb 20;13(2):e0192934. doi: 10.1371/journal.pone.0192934 (PMC5819793; doi:10.1371/journal.pone.0192934)
Supplement: S1 Table — (DOCX) [file pone.0192934.s001.docx]

**S1 Table. Correlations between MENQOL and PSQI scores by menopausal status**

| PSQI items | **MENQOL** | | | |  |
| --- | --- | --- | --- | --- | --- |
|  | **Vasomotor** | **Psychosocial** | **Physical** | **Sexual** | |
| Premenopause |  |  |  |  | |
| Subjective sleep quality | 0.136^*^ | 0.300^**^ | 0.278^**^ | 0.076 | |
| Sleep latency | 0.050 | 0.149^*^ | 0.139^*^ | -0.006 | |
| Sleep duration | 0.095 | 0.030 | 0.077 | 0.119 | |
| Habitual sleep efficiency | 0.031 | -0.016 | 0.062 | -0.024 | |
| Sleep disturbances | 0.134^*^ | 0.154^*^ | 0.163^*^ | 0.052 | |
| Use of sleep medication | -0.042 | 0.053 | -0.023 | -0.066 | |
| Daytime dysfunction | 0.163^*^ | 0.288^**^ | 0.239^**^ | 0.004 | |
| Total PSQI score | 0.181^*^ | 0.314^**^ | 0.305^**^ | 0.086 | |
| Perimenopause |  |  |  |  | |
| Subjective sleep quality | 0.142^*^ | 0.234^*^ | 0.257^**^ | 0.189^*^ | |
| Sleep latency | 0.277^**^ | 0.179^*^ | 0.262^**^ | 0.130 | |
| Sleep duration | 0.007 | 0.036 | 0.131 | -0.016 | |
| Habitual sleep efficiency | -0.001 | 0.000 | -0.034 | 0.019 | |
| Sleep disturbances | 0.266^**^ | 0.173^*^ | 0.288^**^ | 0.174^*^ | |
| Use of sleep medication | 0.196^*^ | 0.167^*^ | 0.162^*^ | 0.066 | |
| Daytime dysfunction | 0.109 | 0.191^*^ | 0.238^**^ | 0.129 | |
| Total PSQI score | 0.213^*^ | 0.222^*^ | 0.318^**^ | 0.139^*^ | |
| Postmenopause |  |  |  |  | |
| Subjective sleep quality | 0.090 | 0.322^**^ | 0.229^*^ | 0.149 | |
| Sleep latency | 0.310^**^ | 0.260^*^ | 0.302^**^ | 0.133 | |
| Sleep duration | 0.204^*^ | 0.240^*^ | 0.152 | 0.101 | |
| Habitual sleep efficiency | 0.251^*^ | 0.159 | 0.123 | -0.020 | |
| Sleep disturbances | 0.311^**^ | 0.291^**^ | 0.281^*^ | 0.165^*^ | |
| Use of sleep medication | 0.014 | 0.001 | -0.091 | 0.020 | |
| Daytime dysfunction | 0.115 | 0.314^**^ | 0.251^*^ | 0.114 | |
| Total PSQI score | 0.322^**^ | 0.421^**^ | 0.332^**^ | 0.171^*^ | |

^*^*P* < 0.05, ^**^*P* < 0.001.
